# A Gem Dedicated to That Rabelias of the Profession

**Published:** 1895-03

**Authors:** 


### A GEM, DEDICATED TO THAT RABELAIS OF THE PROFESSION, T. C. M.

(*Cincinnati Lancet-Clinic.*)

During the last two years our old war pensioners have been put to serious inconvenience, and great loss in some cases, by the exactions of the indefatigable Hoke Smith, and I want to relate an incident in connection with a case of this kind.

Captain K——— was threatened with a 50 per cent. reduction of his pension, and was ordered before the pension board to sustain the existence of his long-continued disabilities. Then he was ordered to undergo examination by a civil surgeon, then back to the board again. Then he was asked to get some general evidence from his family physician, and also to get the affidavits of two or three other physicians to substantiate the fact that he had an inguinal hernia, in connection with a chronic diarrhœa. The Captain listened to the learned dissertations of the surgeons in their differential diagnosis of hydrocele and hernia until he got bewildered, and finally penned Hoke Smith the following epistle:

DEAR MR. SMITH—I have been sent around from pillar to post, from poor doctors to worse ones, and I want to tell you a story. One beautiful summer evening a couple of young lovers might have been seen lounging in close proximity to each other, beneath a budding apple tree, tenderly caressing each other. The young lady was clad in those clinging, expensive robes, which betokened that she was the child of rich, but respectable parents, and the young man also had the earmarks of prosperity. Suddenly from out the stillness came the soft, sweet voice of the maiden, who said: "Algernon, I discover that your right kidney hangs a little lower than the left, doesn't it?" Mr. Smith, please send me to some doctor who knows the difference between a kidney and a testicle.

